# Patient directed self management of pain (PaDSMaP) compared to treatment as usual following total knee replacement: study protocol for a randomized controlled trial

**DOI:** 10.1186/1745-6215-13-204

**Published:** 2012-11-05

**Authors:** Simon Donell, Katherine Deane, Louise Swift, Garry Barton, Paula Balls, Clare Darrah, Richard Gray

**Affiliations:** 1 Norfolk and Norwich University Hospitals NHS Foundation Trust, Colney Lane, Norwich, NR4 7UY, UK; 2 Faculty of Medicine and Health Sciences, University of East Anglia, Norwich Research Park, Norwich, NR4 7TJ, UK

**Keywords:** Randomized controlled trial, Analgesia, Patient directed, Total knee replacement

## Abstract

**Background:**

In 2009, 665 patients underwent total knee replacements (TKRs) at the Norfolk and Norwich University Hospitals NHS Foundation Trust (NNUH), representing nearly 1% of the national total. Pain control following the operation can be poor, and this can cause poor mobilization and potential long-term adverse events. Although high levels of pain are not associated with patient dissatisfaction, brief periods of pain may lead to neuronal remodeling and sensitization. Patient controlled oral analgesia (PCOA) may improve pain relief; however, the evidence to date has been inconclusive. Patient directed self management of pain (PaDSMaP) is a single center randomized controlled trial, which aims to establish if patient self-medication improves, or is equivalent to, treatment as usual and to create an educational package to allow implementation elsewhere.

**Methods/design:**

Patients eligible for a TKR will be recruited and randomized in the outpatient clinic. All patients will undergo their operations according to normal clinical practice but will be randomized into two groups. Once oral medication has commenced, one group will have pain relief administered by nursing staff in the usual way (treatment as usual; TAU), whilst the second group will self manage their pain medication (patient directed self management of pain; PaDSMaP). Those recruited for self-medication will undergo a training program to teach the use of oral analgesics according to the World Health Organization (WHO) pain cascade and how to complete the study documentation. The primary endpoint of the trial is the visual analogue scale (VAS) pain score at 3 days or discharge, whichever is sooner. The follow-up time is 6 weeks with a planned trial period of 3 years. The secondary objectives are satisfaction with the management of patient pain post-operatively whilst an inpatient after primary TKR; overall pain levels and pain on mobilization; satisfaction with pain management information provided; global outcomes, such as quality of life (QOL) and activities of daily living (ADLs); time to mobilization and whether time to mobilization is associated with frequency of adverse events, improvements in QOL, ADLs and pain at 6 weeks after the operation; incidence of adverse events; quantity and type of pain medications used whilst an inpatient; the acceptability of PaDSMaP and/or TAU protocols for patients and the healthcare professionals involved in their care; to investigate the health-related costs associated with a PaDSMaP system; and to estimate the cost-effectiveness of PaDSMaP compared to TAU.

**Trial registration:**

Current Controlled Trials ISRCTN: 10868989

## Background

In the four years to 2007 to 2008, the number of primary total knee replacements (TKRs) increased by 163% to 73,455 in England and Wales. These figures are expected to continue increasing owing to the ageing demographic of the UK population [1] and the ability of TKRs to be conducted with good outcomes in the very elderly (>80 years of age) [2]. In 2009, 665 patients underwent TKR operations at the Norfolk and Norwich University Hospitals NHS Foundation Trust (NNUH), representing nearly 1% of the national total.

After a TKR operation, patients experience substantial pain but there is debate on the best way to manage it [3,4]. Poorly controlled post-operative pain can have significant and potentially long-term adverse effects on patients. Pain can prevent early mobilization, which substantially raises the risk of patients developing deep vein thromboses [5]. Even brief intervals of acute pain can induce long-term neuronal remodeling and sensitization (‘plasticity’), chronic pain, and lasting psychological distress [6,7]. However, paradoxically, high levels of pain are not associated with patient dissatisfaction with their pain management [8].

The World Health Organization (WHO) introduced the concept of the analgesic ladder [9]. Under this regimen analgesics are introduced as follows:

• Step one: Non-opioid analgesics. For example aspirin, paracetamol, non-steroidal anti-inflammatory drugs (NSAIDs). If anticipation of pain can be abolished, it may not be necessary to step up to opioids. Give non-opioid regularly and use adjuvants if necessary.

• Step two: Mild opioids. For example codeine, dihydrocodeine; with or without non-opioid.

• Step three: Strong opioids. For example morphine (Oramorph); with or without non-opioid.

The WHO guidelines for pain management state that the evidence now suggests that pain can be best managed when drugs are taken ‘by the clock’ [9]. However, any breakthrough pain should also be treated promptly and effectively. Therefore adequate monitoring of pain levels is a key principle of pain management and all participants in this trial will be encouraged to monitor their pain at least three times a day. Although the WHO pain control ladder was originally designed for chronic cancer pain, its principles have since been applied to acute pain and have been adapted to accommodate modern analgesics, including adjuvant analgesics [10].

It is further proposed that a patient’s level of pain and satisfaction with care may be influenced by who delivers pain control medications, whether it is patient directed (patient directed self management of pain; PaDSMaP) or under nurse control (treatment as usual; TAU). The ‘by the clock’ analgesia regimen may be more easily followed in patient controlled oral analgesic (PCOA) protocols. It is proposed that PCOA may also allow patients to vary their analgesia more easily according to pain and activity levels, for example in advance of mobilization. In addition, since pain is a combination of tissue damage and emotional state, it is hypothesized that being in control via self-medication may reduce the emotional component of pain. It is known that psychological resilience and preparedness make it easier to control pain [11,12].

However, there is limited and divergent evidence for the efficacy and acceptability of PCOA. Two studies by Striebel [13,14] claimed PCOA increased patient satisfaction and pain control. In contrast, a recent pilot study of PCOA provided on just the second post-operative day after TKR showed no significant differences between the PCOA and nurse controlled analgesia groups [15].

Patient understanding about analgesia is critical for adherence, since it has been found that patients take fewer analgesics than prescribed post-operatively, even if they report a high level of pain [16,17]. Patients on patient controlled analgesia (PCA, usually via infusion) reported controlling the use of analgesics to balance side-effects, self-image and pain [18]. Thus, the use of analgesics by patients is not as simple as ‘just’ controlling pain levels.

Post-operative pain management at the NNUH will be given by an initial ‘by the clock’ approach to medicating, to prevent pain before it resurfaces. Ongoing monitoring of the pain will take place to ensure there is no breakthrough pain and then either additional ‘as needed’ doses or downward titrations of the initial dosage, depending on pain levels, can be administered. This type of proposed schedule is consistent with the flexibility recommendations of the Agency for Healthcare Research and Quality (AHRQ) [19].

Therefore, the information provided to participants should cover issues of importance to them [20], including expectations of the pain experience, the analgesic plan, the importance of a ‘by-the-clock’ approach, management of analgesic side-effects and non-pharmacological methods of pain control [4,18].

This study’s PaDSMaP intervention addresses the key aim of the Darzi Report [21]: ‘An NHS that gives patients and the public more information and choice, works in partnership and has quality of care at its heart’.

### Primary objective

To investigate if PaDSMaP reduces pain at 3 days or on discharge, whichever is sooner, after primary TKR operation compared to TAU.

### Secondary objectives

To investigate whether the PaDSMaP and TAU groups differ in terms of:

• Patient satisfaction with the management of their pain post-operatively whilst an inpatient after primary TKR.

• Overall pain levels and pain on mobilization.

• Satisfaction with pain control.

• Satisfaction with pain management information provided.

• More global outcomes, such as quality of life (QOL) and activities of daily living (ADLs).

• Time to mobilization, and whether time to mobilization is associated with frequency of adverse events, and improvements in ADLs, QOL and pain at 6 weeks after the operation.

• Incidence of adverse events.

• Quantity and type of pain medications used whilst inpatients.

Additional secondary objectives include: to investigate the acceptability of the PaDSMaP and TAU protocols for patients and the healthcare professionals involved in their care; to investigate the health-related costs associated with a PaDSMaP system; and to estimate the cost-effectiveness of PaDSMaP compared to TAU.

## Methods

### Ethics approval

This study has been approved by the Cambridgeshire 1 Research Ethics Committee approval Ref: 10/H0304/52. The study will be conducted in full conformity with the current revision of the Declaration of Helsinki (last amended October 2000, with additional footnotes added 2002 and 2004), and in full conformity with relevant regulations and the International Conference on Harmonisation of Technical Requirements for Registration of Pharmaceuticals for Human Use (ICH) Guidelines for Good Clinical Practice, CPMP/ICH/135/95, July 1996.

A research nurse from the study team will meet with the patient at the pre-operative assessment clinic (2 to 3 weeks prior to operation) and make a final check on trial eligibility. Written and verbal versions of the participant information leaflet will then be presented detailing the exact nature of the study. In particular, the research nurse will explain the randomized allocation element of the trial. It will be made clear that the patient is free to withdraw from the study at any time for any reason without prejudice to future care, and with no obligation to give the reason for withdrawal. The patient will be allowed as much time as they wish to consider the information and ask questions. If required, the research nurse will facilitate opportunities to question other members of the research team, the patient’s general practitioner (GP) or other independent parties, to decide whether the patient can participate in the study. This may mean that the research nurse has to arrange a subsequent home visit at a later date after prior agreement and arrangement with the patient.

Written informed consent will be obtained by means of a participant dated signature and dated signature of the research nurse. These signatures will be obtained on the latest approved version of the informed consent form, before any study-specific procedures are performed. A copy of the signed informed consent will be given to the participant and a copy will be retained by the Trial Office at the University of East Anglia (UEA). The original signed form will be sent to the participant’s GP.

For those participants recruited into the qualitative interview aspect of the trial, a further information sheet will be given and a consent form will subsequently be completed and signed. As above, a copy will be given to the participant and a second copy will be retained by the Trial Office at the UEA.

All data will be handled in accordance with the Data Protection Act 1998, which requires data to be anonymized as soon as it is practical to do so. Each participant will have a case record file containing copies of consent forms, completed measures and demographic information. Case record files will be kept in a locked cabinet within a locked room at NNUH. Only the chief investigator (RG), the project lead (KD) and the research nurse will have access to these. Both RG and KD have extensive experience of clinical trials, and RG and KD are trained in the ICH and WHO Good Clinical Practice Standards for Clinical Trials.

The trial staff will ensure that the participants’ anonymity is maintained. The participants will be identified only by initials and a participant ID number on case report forms and electronic databases. All documents will be stored securely and will only be accessible by trial staff and authorized personnel.

### Trial design

#### Summary of trial design

This study uses a prospective, randomized, parallel-group design to compare PaDSMaP to TAU for patients undergoing a primary unilateral TKR at a teaching hospital, the NNUH. The analysis of clinical outcomes will be blinded. It will not be possible to blind the health economic data analysis.

One hundred and forty-four patients who have just undergone TKR will be randomly assigned to the PaDSMaP or TAU groups pre-operatively. This study will investigate whether PaDSMaP improves levels of pain at 3 days or discharge, whichever is sooner, after a TKR operation, compared to TAU. It will also compare the two groups up to 6 weeks after the operation for pain levels, satisfaction with the control of pain, return to walking and normal activities, as well as any further problems. We will also interview a sample of 12 patients (six patients from each group) and 12 healthcare professionals involved in their care, to explore their experiences of the PaDSMaP and TAU protocols. Finally, we will measure the costs of PaDSMaP compared to those of TAU.

In addition, the patients entered into this study will form a group that will be followed up in standard nurse-led remote follow-up clinics for up to 5 years, to determine the impact of pre-operative characteristics (such as anxiety, depression, pain levels) and levels of post-operative pain control on the long-term success or failure of TKRs, as defined by the development of chronic pain, return to normal activities and impact on QOL.

#### Primary and secondary outcome measures

The primary outcome for PaDSMaP is pain levels at discharge or after 3 days, whichever is sooner, after primary unilateral TKR in patients, as measured by a 10 cm continuous visual analogue scale (VAS).

Secondary outcome measures for the patients comprise:

• Pain levels (pre-operative assessment clinic baseline (2 to 3 weeks prior to operation), Days 1 to 3 and 6 weeks post-operatively).

• Pain after mobilization (3 times a day for Days 1 to 3 post-operatively).

• Satisfaction with pain levels (Days 1 to 3 and 6 weeks post-operatively).

• Satisfaction with Information about Medicines Scale (SIMS) (Day 3 and 6 weeks post-operatively).

EuroQoL EQ-5D questionnaire (EQ-5D) (pre-operative assessment clinic baseline, 6 weeks post-operatively).

• Oxford Knee Score (OKS) (pre-operative assessment clinic baseline, 6 weeks post-operatively).

Time to mobilization (inpatient notes).

• Adverse events (Days 1 to 3 and 6 weeks post-operatively).

• Medication usage (pre-operative assessment clinic baseline, Days 1 to 3 and 6 weeks post-operatively).

• A health resource use questionnaire (pre-operative assessment clinic baseline, 6 weeks post-operatively).

• Qualitative evaluation of patients’ and health professionals’ experiences (five patients from each group 6 weeks post-operatively and ten healthcare professionals after trial protocol has finished).

See Table 1.

**Table 1:** Patient outcome measures time points

**Patient outcomes**	**Baseline**	**Post-operative Day 1**	**Post-operative Day 2**	**Post-operative Day 3 or discharge, whichever is sooner**	**Post-operative 6 weeks**
Pain VAS	x	Breakfast, lunch, supper	Breakfast, lunch, supper	Breakfast, lunch, supper	x
Pain VAS after mobilization		x	x	X	
Satisfaction with pain levels^1^	x	x	x	X	x
SIMS^2^				X	x
EQ-5D	x				x
OKS	x				x
Time to mobilization		x	x	X	
Adverse events	x	x	x	X	x
Medication usage^3^	x	x	x	X	x
Health resource use questionnaire	x				x
Qualitative interviews^*^					x

^*^Subsample of five patients from each group; ^1^Completed at lunchtime and at discharge; ^2^Completed at discharge; ^3^All medications in last 24 hours, including anaesthetic agents (except inhaled anaesthetics). VAS, visual analogue scale; SIMS, Satisfaction with Information about Medicines Scale; EQ-5D, EuroQoL EQ-5D questionnaire; OKS, Oxford Knee Score.

#### Trial participants

The study is aimed at patients who are about to undergo a primary unilateral TKR. We will recruit patients referred to the orthopaedic clinics for TKR at the NNUH.

##### Inclusion criteria

• All adult patients aged over 18 years undergoing a primary TKR operation.

• Meet the NNUH self management of pain criteria.

• Are expected to require standard step 1 to 3 oral analgesics post-operatively

• Post-operatively, patients must be awake and breathing independently, able to answer questions and follow commands to continue in the protocol.

• Are English speaking and literate. (We expect the patient participants to be able to read the information sheet for the PaDSMaP protocol and fill in a number of self-assessments. Less than 1% of the population over the age of 50 years, which are the usual candidates for TKRs, are non-English speaking in our catchment area.)

• Patients may have received regional blocks or epidural analgesia and will start PaDSMaP or TAU as soon as they begin oral analgesia.

##### Exclusion criteria

• Expected to require intensive care.

Known, or suspected to be, opioid tolerant or dependent.

• Regular users of any modified release opiate preparation during the 2 weeks prior to TKR.

• Recent history of drug or alcohol abuse.

• Lack competence to consent by reason of dementia or any other reason.

• Any patient who does not self-administer at home.

These inclusion and exclusion criteria are in line with the NNUH’s drug self-administration policy.

#### Study procedures

##### Recruitment

Patients eligible for a primary TKR will be given an information sheet and consent form at the outpatient clinic where the patient is added to the TKR waiting list (approximately 6 weeks prior to operation). In addition, any potential participant who is missed from this clinic, but is placed on the TKR waiting list, will also be sent an information sheet and consent form by post.

A phone call from the research nurse will be made to eligible patients approximately 1 week prior to the pre-operative assessment clinic. The purpose of this phone call is to determine if the patient is interested in participating in the PaDSMaP study. The phone call will not be regarded as confirmation of patient consent to participate in the study; consent will be taken during the clinical appointment. The purpose of the call is to ensure that the research nurse is available to appropriately conduct consent and carry out baseline measures and education on the PaDSMaP protocol, as appropriate.

##### Recruitment and informed consent for interviews

Six patients from each group will be purposively sampled to participate in interviews to assess the acceptability of the PaDSMaP and TAU protocols. The patients will be purposively selected according to pre- and post-operative characteristics that may have had an impact on their experience of the pain control protocols (for example age, pain levels after TKR, length of stay).

The selected patients will be sent separate information sheets and consent forms for this section of the research protocol at least 1 week prior to their 6 week follow-up outpatient appointment by the research nurse. At this appointment the research nurse will explain the interview process, the arrangements for the interview and ensure the patient understands the patient information leaflet for interview detailing the exact nature of the interviews. Again it will be made clear that the patient is free to withdraw from the study at any time for any reason without prejudice to future care, and with no obligation to give the reason for withdrawal. The patient will be allowed as much time as they wish to consider the information and ask questions. If consent is given, the research nurse will organize an appointment for the interview. The interview can take place at the patient’s home or in a private room at the hospital, whichever is the patient’s preference. Should the patient withdraw from the interview at the time it is undertaken, the data will not be used. Once an interview transcript has been received by the patient for checking they will then have a 2 week period to withdraw from this section of the study, or it will be presumed that that the transcript can be used.

Twelve healthcare professionals, representative of the team that looks after patients after a TKR, will also be purposively selected to participate in interviews to investigate their views on the PaDSMaP and TAU protocols. These interviews will be held after the majority of patients we aim to recruit have passed through the PaDSMaP protocol. The healthcare professionals will be sent information sheets and consent forms. The research nurse will then contact them to ask if they would be willing to participate in the interview.

If they indicate they are willing, an appointment will be made to meet with the research nurse during working hours in a private room at the hospital. The research nurse will explain the interview process, the arrangements for the interview and ensure the healthcare professional understands the healthcare professional information leaflet detailing the exact nature of the interviews. It will be made clear that the healthcare professional is free to withdraw from the interview at any time for any reason and with no obligation to give the reason for withdrawal. If written consent is provided, the interview will proceed. Withdrawal from the study will be on the same basis as the patient interviews.

##### Assessments

When consent has been obtained the research nurse will collect baseline data from patients. The research nurse will be trained in administering questionnaires in a standardized way. The majority of the outcomes will be self-completed by the patients. The research nurse will regularly visit the orthopaedic wards to encourage completion of the questionnaires by patients in both arms of the trial. The following information will be obtained.

### Primary outcome

#### Pain VAS (visual analogue scale)

Pain levels will be measured using a 10 cm continuous VAS bounded by the phrases ‘no pain’ and ‘worst possible pain’. [22] Patients will mark with a cross to indicate where their pain level over the previous 4 hours lies. This will be measured at the end of Day 3 post-operatively or at discharge, whichever is sooner.

### Secondary outcomes

#### Pain VAS

Pain VAS for the previous 4 hours will also be measured at a variety of time points (at baseline, post-operative Days 1 to 3 at breakfast, lunch and supper, and at the 6 week follow-up clinic; see Table 1), to determine the pattern of pain pre- and post-operatively. We will also measure current pain levels on a 10 cm VAS after mobilization with the physiotherapist. Finally, patients will be asked to note if pain was sufficient to wake them during the night (Yes/No answer).

#### Patient satisfaction with pain control

Patients will indicate their satisfaction with the control of their pain on a five-point Likert-type scale. They will indicate the degree to which they agree or disagree with statements about their satisfaction with the control of their pain.

#### Satisfaction with Information about Medicines Scale (SIMS)

Patients will indicate their satisfaction with the information they received on a validated 17-item tool, designed to assess the extent to which patients feel they have received enough information about prescribed medicines [23].

#### Oxford Knee Score (OKS)

The OKS was designed for the assessment of TKR outcomes [24]. The score is derived from a 12-item questionnaire which is self-administered by the patient. Importantly, the questions were designed with input from patients themselves in order to try to ensure that the information derived was as valid and sensitive as possible. This score appears to be simple and reliable, as well as being sensitive to clinically-important changes over time within one patient. This score is also part of the patient reported outcome measures (PROMs) package [25].

#### EuroQoL EQ-5D questionnaire (EQ-5D)

The EQ-5D [26] is a widely used generic utility measure, which is used to characterize current health states. It consists of five dimensions, which each have three levels and a VAS. This measure will be used in the cost-effectiveness analysis (Brooks 1996), where responses will be sought at baseline and 6 weeks post-operatively. This will subsequently enable the quality-adjusted life year (QALY) gain associated with the intervention to be calculated. The EQ-5D is being used as part of the PROMs package nationally to track outcomes of TKRs [24].

#### Time to mobilization

The information regarding the time and day that the patient is able to stand up and transfer from bed to chair will be taken from the notes by the research nurse onto the case report form.

#### Adverse events

Adverse events will be identified by self-completed reports from patients and from patient notes by the research nurse on the case report form. These will be reported regularly and discussed with the clinical lead (SD), the Data Monitoring and Ethics Committee (DMEC) chair (Dr Sanders) and, where appropriate, with the governance lead for the Trust. Adverse events will be recorded from the point patients are entered into the study (at randomization in the pre-operative assessment clinic) to the point at which they leave the study (the 6 week follow-up clinic).

#### Medication usage

All medications that are used by the participants in the study will be documented. At baseline, all medications and dosages currently taken by the patient will be recorded, particularly those used for pain relief. During the hospital inpatient stay participants in the PaDSMaP group will be asked to note the type of pain relief medication they took, the dose and the exact time at which they took it on the inpatient prescription chart. Digital illuminated clocks will be provided in the drug boxes for the PaDSMaP group. Nurses looking after patients in the TAU group will be asked to note the exact time of delivery of the analgesic drugs on the inpatient prescription chart. At the 6 week follow-up, patients will be asked to report their current medication usage.

#### Healthcare related costs

In line with guidance from the National Institute for Health and Clinical Excellence (NICE) [27], costs will first be calculated from the perspective of the National Health Service (NHS) and personal social services, and encompass those costs that are potentially related to the intervention in question. Costs of the intervention, for example training the nurses, training the patients and provision of locked medication boxes will be recorded. Levels of resource use incurred in the 6 weeks post-randomization will be recorded, including use of medications, pharmacy costs, the length of original hospital stay, input by healthcare professionals in hospital (for example ward nurse time, physiotherapists) and subsequent outpatient or GP visits post-discharge. Unit costs will subsequently be assigned to each of these resource items.

### Baseline characterization of the population

The following measures regarding the character of the recruited population will also be collected at baseline. These will inform assessments of the generalizability of the study, the success of the randomization process and may help to identify some potential confounding factors:

• Age (years).

• Gender.

• Ethnicity.

• Whether there will be someone to assist the patient on discharge (for example spouse or carer).

• Whether the residence the patient will be discharged to has stairs.

• Level of education.

• Socioeconomic status (estimated using participants’ postcodes).

• Body mass index (BMI, from notes).

• Duration of knee pain.

• Current level of knee pain (VAS).

• Previous experience of orthopaedic surgery (what and when).

• Expectation of level of pain post-operatively at Days 1 to 3 and week 6 (VAS).

• Medication profile (type of medications, dosage level and frequency).

• Comorbidities.

• Other musculoskeletal problems.

• Surgical procedures undertaken in TKR (surgical procedure questions).

• Hospital Anxiety and Depression Scale (HADS).

• Beliefs about Medications Questionnaire (BMQ).

#### Surgical procedure questions

Surgical procedure questions will be gathered as part of standard practice for the National Joint Registry (NJR) [28]. They will allow us to determine if the type of surgical procedure had a bearing on patients’ pain and recovery.

#### Hospital Anxiety and Depression Scale (HADS)

The HADS [29] identifies anxiety disorders and depression, and has been widely used and validated [30]. Anxiety and depression have been identified as factors that can affect post-operative pain and recovery [11]. Should the HADS identify depression, this information will be fed back to the patient immediately along with one of two information sheets that recommends the patient to contact their GP (moderate/severe depression) or refers the patient to relevant self-help websites (mild depression). A letter will also be sent (with the patient’s knowledge) to their GP informing them that the patient has been identified as having depression and informing them of what recommendations the research team has made to the patient.

#### Beliefs about Medications Questionnaire (BMQ)

This BMQ [31] will be used to measure patients’ attitudes and beliefs toward medication. Since these patients will be prescribed multiple drugs the general version of the measure will be used. Each item is rated on a five-point Likert-type scale ranging from ‘strongly disagree’ (1) to ‘strongly agree’ (5). The questionnaire has four sections that evaluate attitudes about:

1. General harm (G-H) that is, the intrinsically harmful properties of medications (four questions, such as ‘most medicines are addictive’).

2. General overuse (G-O) of medications by healthcare professionals (four questions, such as ‘Doctors use too many medicines’).

3. General sensitivity (G-S) to adverse events from medications (five questions, such as ‘my body is very sensitive to medicines’).

4. General benefit (G-B) that is, the intrinsically beneficial properties of medications (four questions, such as ‘in most cases the benefit of medicines outweigh the risks’).

#### Randomization

The research nurse will check patient eligibility and consent against a checklist. Authorized research nurses will each be allocated a six-digit personal identification number (PIN) for use when randomizing patients. The research nurse will telephone the independent randomization service at the Clinical Research and Trials Unit (CRTU) at the UEA. The system will request the PIN and will only proceed if a valid PIN is entered via the telephone keypad.

On entry of a valid PIN, the system will generate a unique study code and randomly allocate the patient to either the PaDSMaP or TAU arm of the trial. The study code and allocation will be reported back to the caller verbally and will also be sent in a confirming email to: a) the caller, b) other nominated trial staff and c) the trial database manager. The study code and allocation will be stored in the trial database on the secure CRTU server at UEA.

A computer generated randomization list will allocate patients in a 1:1 ratio to PaDSMaP or TAU groups. Randomization is unstratified. To ensure a reasonably even distribution of patients in the two arms throughout the course of the trial, patients will be allocated in randomly distributed blocks of four and six.

At the pre-operative assessment clinic, patients that have expressed an interest in participating in the PaDSMaP study will go the CRTU after their clinic appointment. At CRTU they will be consented and randomized. The baseline assessments will be conducted and patients instructed on how to complete the trial inpatient outcome measures. It is anticipated that this will take about 1.5 hours in total.

### Interventions

#### TAU

Patients in the TAU group will receive the standard general information sheet and DVD outlining the TKR operation and recommendations for both pre- and post-operative exercises [32].

The study is a pragmatic trial and as such normal variations in pre- or post-operative anaesthetic are part of what will be examined. The patient’s initial post-operative analgesic care will be under the direction of the anaesthetists and this normally lasts for 12 to 24 hours post-operatively. Oral analgesia will be initiated prior to pain resurfacing after, for example, the epidural has worn off. Patients’ oral post-operative analgesia on the wards will be dispensed as usual by the nursing team to the TAU group.

We expect that the majority of patients recruited to this study will receive the Modified Caledonian Technique (MCT) Norwich Enhanced Recovery Program (NERP). This program aims to enhance the recovery of patients having primary knee replacements by a multimodal program, which facilitates early mobility and discharge. The NERP focuses on the provision of safe and effective analgesia with minimal side-effects, which then enable early mobility. Of note, gabapentin is included because it is an opioid-sparing medication and reduces opioid-related side-effects [33].

Centers in the UK and abroad adopting the enhanced recovery program have reduced patients’ length of stay significantly. It is likely that the exact details of the NERP will develop over the course of the trial. It should also be noted that individual patients will always be offered the most suitable protocol for their particular circumstances, so some patients will not follow the NERP protocol. Again this does not prove an obstacle, since the study is a pragmatic trial and variations in care are part of what is to be examined.

Enhanced recovery programs are multimodal and focus on the following elements:

1. Educating GPs and community support services.

2. Patient education and support.

3. Pre-operative physiotherapy, and occupational therapy assessment and education.

4. Pre-operative anaesthetic and surgical assessment.

5. Utilizing surgical and anaesthetic techniques, which facilitate early mobility.

6. Excellent post-operative analgesia allowing early patient mobilization.

7. Intensive post-operative physiotherapy.

8. Early discharge with appropriate back-up and follow-up.

#### Exemplar analgesic protocol

##### In pre-operative period

• Gabapentin 300 mg orally (2 hours before operation or can be given the night before).

##### At commencement of the anaesthetic process

• Single shot spinal 2.5 to 3.0 ml bupivacaine plus total continuous infusion (TCI) of propofol for sedation (or a light general anaesthetic if anaesthetist prefers).

• Ondansetron 4 mg intravenous (IV).

• Paracetamol 1 g IV.

• Dexamethasone 8 mg IV.

• Diclofenac 75 mg IV, if tolerated.

• Tranexamic acid (given to minimize blood loss; has 90 minute half-life so given just before tourniquet goes down for TKR).

• No opiates.

• No urinary catheter.

• Limit fluids to approximately 1 litre intra-operatively, if possible.

##### At end of operation

• Surgeon infiltrates joint space with 150 to 200 ml of 0.2% ropivacaine .

• Surgeon places clearly labeled catheter into the joint space (periarticular catheter).

• Infusion set up with McKinley pump of ropivacaine 0.2%, 200 ml.

• Give 20 ml bolus and set pump at 5 ml per hour.

##### Post-operatively

• Gabapentin 300 mg twice a day for 5 days.

• Paracetamol 1 g orally four times a day.

• Ibuprofen 400 mg three times a day, if patient is able to tolerate.

• Oxycodone (OxyContin) 10 to 20 mg twice a day for 3 to 5 days.

• Morphine (Oramorph) 5 to 20 mg as needed up to every 2 hours for breakthrough pain.

• Ondansetron 4 mg twice a day IV as needed.

• (Intramuscular morphine as needed for escape analgesia is also prescribed.)

#### Exemplar physiotherapy protocol

Providing that the patient’s systolic blood pressure is within 10% of its pre-operative reading, the patient will be mobilized 4 hours after surgery (Day 0). If a patient feels dizzy on attempting to mobilize, or their blood pressure drops to below 80 mmHg systolic, oral ephedrine 30 mg (or intramuscular ephedrine if no oral ephedrine is available) is given and mobilization is attempted again after 30 minutes.

The patient will be mobilized three times on the first post-operative day. Once the patient is stable on crutches without support, they will be encouraged to mobilize independently. The patient will receive physiotherapy three times a day until discharge.

#### Exemplar discharge protocol

If appropriate, the patient will be discharged on Day 3 to 4. The discharge criteria will be:

• Able to mobilize independently.

• Pain is well controlled on oral analgesics.

Patients will be discharged with 14 days of oral analgesics and informed that they must visit their GP for a further prescription. The patient will be telephoned at home on Day 7 post-discharge.

#### Development of the PaDSMaP package

Effective treatment of pain requires individually tailored care pathways and treatment strategies. The PaDSMaP package will be developed by members of the research team in conjunction with relevant clinical and lay experts. This will result in a comprehensive, structured, evidence-based training manual with associated information in a range of appropriate formats, capable of being tailored according to individual needs, and designed for delivery by a trained research nurse and the post-operative ward team. Patients in the TAU group will receive the PaDSMaP patient information sheet, but all other aspects of their care before, during, and after their TKR operation will be TAU.

#### Pre-operative education: PaDSMaP group

Patients will get a short break after the consent process and baseline assessments are completed before they receive training in the PaDSMaP protocol. Patients will receive an information sheet and a DVD, which in addition to the standard information on the operation and pre- and post-operative exercises, will include information on the PaDSMaP self-medication protocol. The research nurse will then take 20 minutes to explain how to self-monitor their pain and medicate it appropriately. Patients will receive protocols outlining how to maximize analgesia, how they can adjust their analgesic levels, and receive information prior to the operation about appropriate pain control and why pain control is important (earlier mobilization, reduction in adverse events, and so on).

The PaDSMaP information sheet covers the issues identified by Kastanias [20] as important to patients regarding post-operative pain management. These will include:

• Expectations of the pain experience itself.

• What the analgesic plan is and what to do if it does not work.

• Other ways of dealing with pain in addition to medicine (relaxation and distraction techniques, ice packs) [4,18].

• Side-effects of analgesics, both what to expect and how to manage side-effects.

#### Post-operative analgesia

Patients in the PaDSMaP group will be in charge of their post-operative oral analgesia. The patients will have a lockable drugs cabinet at their bedside that is easily accessible to them. The drugs cabinet will contain 14 days’ supply of all of the drugs a patient is prescribed for self-medication (all analgesics and the patient’s other usual medications, but excluding any prescribed schedule drugs, for example oxycodone). The drug boxes will be resupplied on a daily basis to ensure 14 days’ supply is available. The usual prescribed regimen will be as for TAU post-operative oral analgesia:

• Gabapentin 300 mg twice a day for 5 days.

• Paracetamol 1 g orally four times a day.

• Ibuprofen 400 mg three times a day, if patient is able to tolerate.

• Oxycodone (OxyContin) 10 to 20 mg twice a day for 3 to 5 days.

• Morphine (Oramorph) 5 to 20 mg as needed up to every 2 hours for breakthrough pain.

Some drugs, such as oxycodone (OxyContin), are not approved for self-medication protocols at NNUH owing to their being scheduled drugs. In the event that a scheduled drug is prescribed, it will be delivered by the ward nurse as part of their usual drug rounds. The scheduled drug(s) will be the only drug(s) that patients receive from the ward nurses; patients will self-medicate with all other drugs.

Patients will be discharged with 14 days’ supply of drugs. This will not usually include morphine (Oramorph) or oxycodone (OxyContin).

The WHO guidelines for pain management state that the evidence now suggests that pain can be best managed when drugs are taken ‘by the clock’ and patients in the PaDSMaP arm will be encouraged to do so. However, patients will also be encouraged to follow a pre-specified protocol to treat breakthrough pain, that is a combination of ‘by the clock’ and ‘as needed’ protocols. Policy documents repeatedly state that adequate monitoring of pain levels is a key principle of pain management and so PaDSMaP patients will be encouraged to monitor their pain hourly. Thus, our plan is an initial ‘by the clock’ approach to medicating (to prevent pain before it resurfaces), ongoing monitoring of the pain to ensure there is no breakthrough pain, and then either additional ‘as needed’ doses or downward titrations of the initial dosage, depending on pain levels. This type of proposed schedule is consistent with the flexibility recommendations of the AHRQ [19].

The ward nurses and pharmacists will also receive appropriate training to support patients who are self managing their pain.

#### Follow-up

Baseline measures of efficacy will be repeated at 6 weeks post-operatively. This time-frame reflects the usual outpatient follow-up clinic time point. The research nurse will ensure completion of all self-assessment scales at this outpatient clinic.

### Analysis

#### Description of statistical methods

Primary and secondary outcomes will be compared between intervention and control groups using t-tests, non-parametric tests or chi-squared and Fisher’s tests, as appropriate. Where differences are observed between outcome measures, demographics and patient characteristics at baseline, adjusted effect sizes will be estimated using linear models.

#### Baseline analyses

To assess external generalizability, demographic and clinical characteristics of participants’ responses at the baseline phase of the study will be compared with participants who are subsequently randomized, and participants who are screened but not randomized. The specific criteria by which participants are excluded from randomization will be tabulated.

#### Number of participants

Seventy-two patients in each arm are sufficient to detect a between-group difference of 0.5 standard deviations in the VAS pain intensity with 80% power using an independent samples *t*-test. Dahlen [34] reported a standard deviation in VAS pain intensity score of 24 points 2 to 5 days after total knee arthroplasty and so assuming a similar variability this represents a difference of about 12 points. Calculations use a significance level of 0.05. A drop-out rate of 10% is expected and accounted for. One hundred and forty-four patients will be randomized in a one-to-one ratio.

Twelve patients from each arm and 12 healthcare professionals will be interviewed to investigate acceptability. The patients will be purposively selected according to pre- and post-operative characteristics that may have an impact on their experience of the pain control protocols (for example age, pain levels after TKR, length of stay). Team members will be selected to represent the spectrum of healthcare professionals that come into contact with patients after a TKR (for example nurses, pharmacists, physiotherapists).

#### Feasibility of target sample size

In 2009, 665 patients had TKR operations at the NNUH. The trial requires recruitment of 144 patients over 24 months, which represents approximately 11% of this population, to meet the requirements of the power calculation. In order to determine if this rate of recruitment was feasible, a mock recruitment was conducted, asking patients that would be eligible for the PaDSMaP trial whether they would consider participation as part of their pre-operative clinic interviews.

Allowing for patients being excluded, the planned recruitment rate is six patients per month over a 24 month period. This target will be reviewed after 6 months.

#### Efficacy analysis

The efficacy of the intervention on the primary outcome (pain VAS) will be assessed by comparing outcomes at discharge or 3 days post-operatively, whichever is sooner, between the two groups. Adjusted estimates will be obtained by identifying baseline variables, which differ between the groups and which are related to the outcome, and incorporating these into a regression analysis.

#### Inclusion in analysis

Both intention to treat (ITT) and per-protocol (PP) analyses will be performed. The ITT analysis set will comprise all patients who have been randomized to each intervention, irrespective of their compliance with the planned course of treatment. This is the primary analysis and will be used for evaluation of all endpoints. The PP set will include patients that have not deviated from the protocol in such a manner that the assessment of efficacy endpoints may be biased. Appropriate adjustments will be made in the statistical analyses for potential confounding factors. These include age, gender, socioeconomic status, level of knee pain pre-operatively, levels of anxiety and depression.

#### Economic evaluation

The aim of the economic evaluation is to assess whether PaDSMaP represents a cost-effective use of scarce NHS resources when compared to TAU.

#### Measuring costs

In line with NICE guidance, costs will be calculated from the perspective of the NHS and personal social services, and encompass those costs that are potentially related to the intervention in question. Thus, for all patients we will monitor the levels of resource use associated with the inpatient stay (including medication use), any re-admission to hospital and other healthcare contacts (for example further therapy, nursing care, and so on).

Patient resource use will be obtained from responses to a health resource use questionnaire at baseline and 6 weeks post-randomization. Appropriate unit costs [35,36] will subsequently be assigned in order to calculate total costs for PaDSMaP and TAU. For the PaDSMaP arm we will estimate the resource use associated with training the research nurse, the ward nurses and pharmacists, and the self-medicating patients. Also, any additional time spent by the ward staff with the PaDSMaP patients related to them facilitating the self-medication regimen will be monitored.

#### Measuring effects

The measure of effectiveness employed in the economic analysis will be measured by the EQ-5D [37]. This is a generic measure of health status designed to compare the benefits of different interventions. It has five dimensions: mobility, self-care, usual activities, pain and anxiety/depression. These will be used to calculate QALYs associated with the intervention and TAU.

#### Potential bias

The subjective nature of the self-report instruments used for evaluation of the intervention is accepted and every effort will be made to minimize potential bias owing to this dynamic. In particular, patients may over- or under-report their health status depending on the trial arm to which they have been assigned.

#### Economic analysis: cost-effectiveness analysis

We will estimate both the mean overall cost and mean overall effect associated with PaDSMaP, compared to TAU. If one of these options is shown to be less costly and more effective than the other, then this would suggest that it ‘dominates’ the other and represents a cost-effective use of scarce resources. Alternatively, the incremental cost-effectiveness ratio associated with PaDSMaP will be estimated and assessed in relation to a range of cost-effectiveness thresholds (for example £20,000 to 30,000 per QALY [27]). The associated level of uncertainty will also be characterized by estimating the cost-effectiveness acceptability curve. Sensitivity analysis will also be undertaken to assess the robustness of conclusions to changes in key assumptions.

### Qualitative evaluation: qualitative investigation of acceptability of the protocols to patients and staff

All patients will be asked to rate their satisfaction with the information provided to them. A phenomenological assessment of the two protocols will be conducted using in depth semi-structured interviews undertaken with a purposively selected subsample of patients (n = 12). In addition, healthcare professionals involved in delivering the intervention will be interviewed to explore their perceptions of the process of using PaDSMaP (n = 12). The aim of these interviews will be to:

• Obtain insights into patients’ and health professionals’ experiences of using the pain relief protocols (PaDSMaP and TAU, as appropriate).

• Consider which elements of the PaDSMaP and/or TAU were perceived as being most and least helpful.

• Explore the participants’ perceptions of the effect that they think PaDSMaP and/or TAU protocols has had on them.

• Uncover any potential barriers and roadblocks to using the PaDSMaP and/or TAU protocols.

• Explore how the PaDSMaP and/or TAU protocols could be refined and enhanced.

An interview schedule will be used for consistency and to ensure coverage of all areas above deemed important. Participants will be prepared for an interview of approximately 30 minutes. Following consent, the first interviews will be conducted by the research nurse and take place at patients’ homes and other appropriate venues (healthcare professionals). All interviews will be audio-recorded, transcribed verbatim and sent to the participants for corroboration. Interviewees will be able to adjust the transcript so that it reflects their views appropriately. Confirmed transcripts from all of the interviews will then be analyzed using the principles of framework analysis to organize the data and identify emerging categories and key themes or concepts using NVivo v.8 [38]. If the intended sample (n = 24) fails to provide a rounded picture and redundancy, additional participants will be interviewed until this is the case.

### Sample frame for selection of participants for qualitative interviews

We will start selecting patients for interview after recruiting 25% of the target number of patients for the study, that is after the first 36 patients. All patients interviewed will have been randomized to the PaDSMaP self-medicating arm of the study. The logic behind the criteria is:

• Gender can influence the relative efficacy of analgesics, may represent differing attitudes to being in pain and to taking medications to control pain.

• Pain expectation may predict post-operative pain levels.

• Length of stay as a representation of a combined measure of pain levels, activity and mobility, and ability to cope relatively independently after the operation.

• Age may influence the activities that one returns to, for example work or retirement. This may influence decisions to have the operation in the first place, expectations of functionality afterwards and activities likely to be undertaken.

Healthcare professionals will be recruited following the patient interviews and will include ward sisters, ward nurses, physiotherapists, pharmacists and members of the pain team. We aim to recruit 12 staff. They will all have treated at least one patient in the PaDSMaP arm and one in the TAU arm. Owing to the difficulty of retaining anonymity of comments in such a small pool of professionals we will refer to staff as either being nurses or allied health professionals (AHPs) in publications.

### Project timetable

The project will take place over 3 years (36 months) including preparation and write-up/dissemination time.

The Research Ethics and Research Governance approvals process will start 6 months before the start of the study to allow all approvals to be secured before the study starts.

Research set-up (minus 6 months to start) will include preparing and submitting ethics and research and development (R&D) applications, refining and finalizing research protocols (minus 6 months to start), recruiting and training the research nurse in the protocol and consent procedures, and setting up research sites (Months 1 to 4).

One hundred and forty-four patients will be identified for the study by the surgical consultants (approximately 6 weeks prior to surgery), and given information sheets and consent forms. They will then be recruited to the study, consented, randomized and baseline information taken by the research nurse at the pre-operative assessment clinic in one 90 minute session per patient (approximately 2 to 3 weeks prior to surgery). Patients allocated to the PaDSMaP arm will also be educated in self-administration of analgesia at this clinic (20 minutes). After surgery, the patients will be given appropriate intervention and assessed daily for 3 days post-operatively or until discharged home, whichever is sooner. The patients will be followed up at the post-operative clinic (6 weeks post-surgery) and the final data will be collected in 30 minute interviews per patient by the research nurse (patients will be recruited in Months 1 to 24).

Thirty minute one-to-one interviews will be conducted by the nurse researcher with a subsample of 12 patients from the PaDSMaP arm and 12 healthcare professionals. Patient interviews will be conducted in their homes after the 6 week post-operative clinic has been conducted (Months 3 to 24) and healthcare professional interviews will occur after the majority of participants have passed through the protocols (Months 20 to 26) in a private room at the hospital.

#### Data entry cleaning and analysis (Months 30 to 36)

Reports and dissemination will include publication of the trial protocol (Month 0), a newsletter to trial participants summarizing the trial’s results, attendance at conferences and publication in relevant peer-reviewed medical journals (Months 34 to 36). The trial will be reported in line with Consolidated Standards of Reporting Trials (CONSORT) 2010 guidelines [39].

Patients in this cohort will continue to be followed up, as is standard practice, and their pain (VAS), ADL (OKS) and QOL (EQ-5D) will be audited up to 5 years post-operatively. (Since this is part of standard practice and all data can be anonymized, no additional consent is required.)

## Discussion

Nurse-originated clinical research leading to a randomized clinical trial is unusual. PaDSMaP is such a trial and although involves orthopaedic patients, it is very much clinical nurse research in emphasis. Whilst the primary outcome for PaDSMaP is pain levels at discharge or after 3 days, whichever is sooner, the secondary outcomes and associated information gained from the study will have important implications for patient care. PaDSMaP is more than just a study looking at the levels of pain relief and comparing self-medicating patients with nurse drug rounds; it is also about understanding the views of all the stakeholders on the acceptability of self-medication. The study aims to show whether patients’ management of pain improves QOL and time to mobilize.

The idea that patients should self-medicate in hospitals raises a number of concerns by staff, especially with ward nurses and pharmacists. The main concerns are patients overdosing on opiate analgesics, as well as their ability to look after themselves following a major operation. In certain specialties where self-medication is already in place, overdosage is not a reported problem. Following an operation, self-medication is delayed until the patient is alert and well enough to do so. The new enhanced recovery programs for surgery include anaesthetic techniques designed to minimize delirium, as well as nausea and vomiting to allow early (day of operation) mobilization. In addition, patients are expected to self-medicate at home, and the ward environment is an excellent place to check on the correct usage and understanding of the medications patients take home.

Regardless of the qualitative outcomes, self-medication may not be acceptable to an NHS with reduced resources, unless it proves itself to be cost-effective, for example in terms of a reduced need for medication, or has a positive effect on length of stay, time to mobilization and adverse effects. The range of measures used in this study aim to give a rounded view on the efficacy of self-administration from both economic, patient and staff perspectives. The qualitative interviews aim to uncover the blocks to implementing this change and will provide valuable insight into the appropriate patient groups where self-medication can be a successful intervention.

Developing the PaDSMaP study has already resulted in the creation of standard operating procedures (SOPs) in self-medicating for the pharmacy and wards, plus instructional booklets on self management of pain and the various drugs involved. This package can be used in other settings where self-administration may be proposed and provides a template for similar packages for other medical conditions.

The trial does have a problem with generalizability. It is a single hospital, single ward study in a mainly rural area with an overwhelmingly English-speaking population. It is also focused on patients undergoing TKR, which was chosen because of the longer length of stay and higher pain levels than total hip replacement or unicompartmental knee replacement. However, it is the nature of the NHS that developments occur all the time. The NERP was introduced soon after the inception of this study and this has shortened the inpatient stay. It is also used for the majority of patients and again this affects the generalizability. NERP standardizes care and so may reduce the advantages (or otherwise) of self-medication. It does, however, reduce the variation in pain management, which was typical only 2 or 3 years ago.

## Conclusions

The single center randomized controlled PaDSMaP trial is designed to look at the efficacy and safety patient self-medicating analgesia following TKR. The trial includes a qualitative element and aims to create a package that can be used to roll out the process in other hospitals.

## Trial status

Recruitment started in July 2011. The study is recruiting to time and target, with recruitment closure anticipated for May 2013.

## Abbreviations

ADLs: activities of daily living; AHRQ: Agency for Healthcare Research and Quality; AHPs: allied health professionals; BMI: body mass index; BMQ: Beliefs about Medications Questionnaire; CONSORT: Consolidated Standards of Reporting Trials; CRTU: Clinical Research and Trials Unit; DMEC: Data Monitoring and Ethics Committee; EQ-5D: EuroQoL EQ-5D questionnaire; GP: general practitioner; HADS: Hospital Anxiety and Depression Scale; ICH: International Conference on Harmonisation of Technical Requirements for Registration of Pharmaceuticals for Human Use; ITT: intention to treat; IV: intravenous; MCT: Modified Caledonian Technique; NERP: Norwich Enhanced Recovery Program; NHS: National Health Service; NICE: National Institute for Health and Clinical Excellence; NIHR: National Institute for Health Research; NJR: National Joint Registry; NNUH: Norfolk and Norwich University Hospitals NHS Foundation Trust; NSAIDs: non-steroidal anti-inflammatory drugs; OKS: Oxford Knee Score; PaDSMaP: patient directed self management of pain; PCA: patient controlled analgesia; PCOA: patient controlled oral analgesia; PIN: personal identification number; PP: per-protocol; PROMs: patient reported outcome measures; QALY: quality-adjusted life year; QOL: quality of life; R&D: research and development; RfPB: Research for Patient Benefit; SIMS: Satisfaction with Information about Medicines Scale; SOPs: standard operating procedures; VAS: visual analogue scale; TAU: treatment as usual; TCI: total continuous infusion; TKR: total knee replacement; UEA: University of East Anglia; WHO: World Health Organization.

## Competing interests

The authors declare that they have no conflicts of interest in this protocol and study.

## Authors’ contributions

All authors were involved in the development of the protocol. CD conceived the research idea. KD was the coordinator and reviewer. LS provided the statistical advice. GB provided the health economics advice. PB provided the qualitative expertise. RG provided nursing, protocol development and trial management expertise. SD provided the orthopaedic perspective and expertise in conducting clinical trials in a secondary care setting. All authors read and approved the final manuscript.
